# Resolving monocytes generated through TRAM deletion attenuate atherosclerosis

**DOI:** 10.1172/jci.insight.149651

**Published:** 2021-10-22

**Authors:** Shuo Geng, Yao Zhang, Ziyue Yi, Ran Lu, Liwu Li

**Affiliations:** 1 Department of Biological Sciences and; 2 Graduate Program of Genetics, Biotechnology and Computational Biology, Virginia Tech, Blacksburg, Virginia, USA.

**Keywords:** Inflammation, Innate immunity

## Abstract

Polarization of low-grade inflammatory monocytes facilitates the pathogenesis of atherosclerosis. However, underlying mechanisms as well as approaches for resolving monocyte polarization conducive to the regression of atherosclerosis are not well established. In this report, we demonstrate that TRIF-related adaptor molecule (TRAM) mediated monocyte polarization in vivo and in vitro. TRAM controlled monocyte polarization through activating Src family kinase c-SRC, which not only induces STAT1/STAT5-regulated inflammatory mediators CCR2 and SIRP-α but also suppresses PPARγ-regulated resolving mediator CD200R. Enhanced PPARγ and Pex5 due to TRAM deficiency facilitated peroxisome homeostasis and reduction of cellular reactive oxygen species, further contributing to the establishment of a resolving monocyte phenotype. TRAM-deficient monocytes propagated the resolving phenotype to neighboring monocytes through CD200R-mediated intercellular communication. At the translational level, we show that TRAM-deficient mice were resistant to high-fat diet–induced pathogenesis of atherosclerosis. We further document that intravenous transfusion of TRAM-deficient resolving monocytes into atherosclerotic mice potently reduced the progression of atherosclerosis. Together, our data reveal that targeting TRAM may facilitate the effective generation of resolving monocytes conducive for the treatment of atherosclerosis.

## Graphical abstract

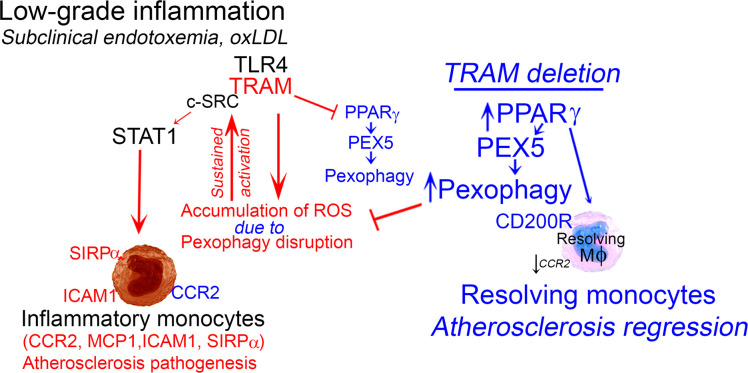

## Introduction

Despite past extensive studies, atherosclerosis and related cardiovascular complications still remain as the leading cause of morbidity and mortality worldwide. The key bottleneck is the limited understanding of complex inflammatory processes, during which reprogrammed low-grade inflammatory monocytes persist and contribute to atherosclerosis pathogenesis (1–3). Inflammatory monocytes exhibit elevated CCR2/CCR5/ICAM-1 (murine intermediate Ly6C^+^; classical Ly6C^++^ inflammatory monocytes, equivalents of human intermediate CD14^+^CD16^+^, classical CD14^+^CD16^–^ inflammatory monocytes) and are the primary innate cells infiltrating atherosclerotic plaques propagating atherosclerosis (4, 5). Inflammatory monocytes contribute to the pathogenesis of atherosclerosis through their enhanced recruitment and retention within the atherosclerotic plaques, as well as their compromised ability of cleaning up necrotic cell debris (4, 6–8). On the other hand, murine Ly6C^lo^ monocytes (human equivalent of CD14^lo^ nonclassical monocytes) may adopt “resolving” features conducive to vascular homeostasis and atherosclerosis regression (9, 10). Precise mechanisms responsible for monocyte polarization associated with atherosclerosis are still not well understood, thus hindering translational efforts in resolving monocyte-mediated inflammatory polarization and the treatment of atherosclerosis.

We established a system that examines history and signal strength–dependent training of low-grade inflammatory monocytes in vitro and in vivo, with the chronic challenge of subclinical low-dose lipopolysaccharide (LPS) (2, 4, 11–13). Subclinical endotoxemia is a well-known risk factor for cardiovascular diseases, due to increased mucosal permeability associated with chronic infection, obesity, aging, high-fat diet, and chronic smoking and drinking (14–17). Both subclinical endotoxemia and oxidized low-density lipoprotein can contribute to the low-grade inflammatory polarization of monocytes at least partially through TLR4, which further signals through 2 potentially competitive Mal/MyD88 or TRIF-related adaptor molecule (TRAM)/TRIF pathways (18–21). Mal/MyD88 pathway preferentially initiates the acute yet transient inflammatory responses and subsequently triggers the late phase of tolerance through orchestrating immune/metabolic adaptations (22, 23). In contrast, emerging studies suggest that the TRAM pathway is preferentially responsible for generating sustained low-grade inflammatory responses, particularly upon stimulation with subclinical super-low-dose endotoxin (2, 12, 24). In the context of atherosclerosis, hematopoietic deletion of TRAM or TRIF, instead of MAL, was shown to reduce the influx of aortic macrophages as well as the severity of atherosclerosis in an animal model (25). However, mechanisms responsible for TRAM-mediated monocyte polarization that favors enhanced infiltration of inflammatory Ly6C^+^ monocytes and the progression of atherosclerosis are still not well understood.

To gain clear insight regarding the nature and underlying mechanisms of polarized inflammatory monocytes, we performed integrative analyses of wild-type and *Tram*^–/–^ monocytes in vitro and in vivo, examining key cell surface inflammatory signatures and relevant intracellular signaling molecules guided by functional relevance to atherosclerosis as well as single-cell RNA-sequencing analyses. We demonstrate that the polarizing effects of subclinical endotoxemia on monocytes were blunted due to TRAM deficiency. At the functional level, we observe enhanced polarization of Ly6C^+^ and Ly6C^++^ inflammatory monocytes with elevated levels of chemokine receptor CCR2 and the suppressor of phagocytosis SIRP-α in TRAM-positive mice, as compared with TRAM-deficient counterparts. TRAM-deficient monocytes not only had attenuated inflammatory responses but also exhibited elevated expression of homeostatic antiinflammatory mediator CD200R due to constitutively active PPARγ. With translational relevance, we show that TRAM-deficient monocytes with elevated CD200R further propagated the resolving characteristics to neighboring monocytes, effectively contributing to the regression of atherosclerosis when transfused into recipient atherosclerotic mice. Together, our data reveal the generation of resolving monocytes through TRAM deletion that are conducive to the treatment of atherosclerosis.

## Results

### TRAM deficiency reduces pathogenesis of atherosclerosis.

**Figure 1:**
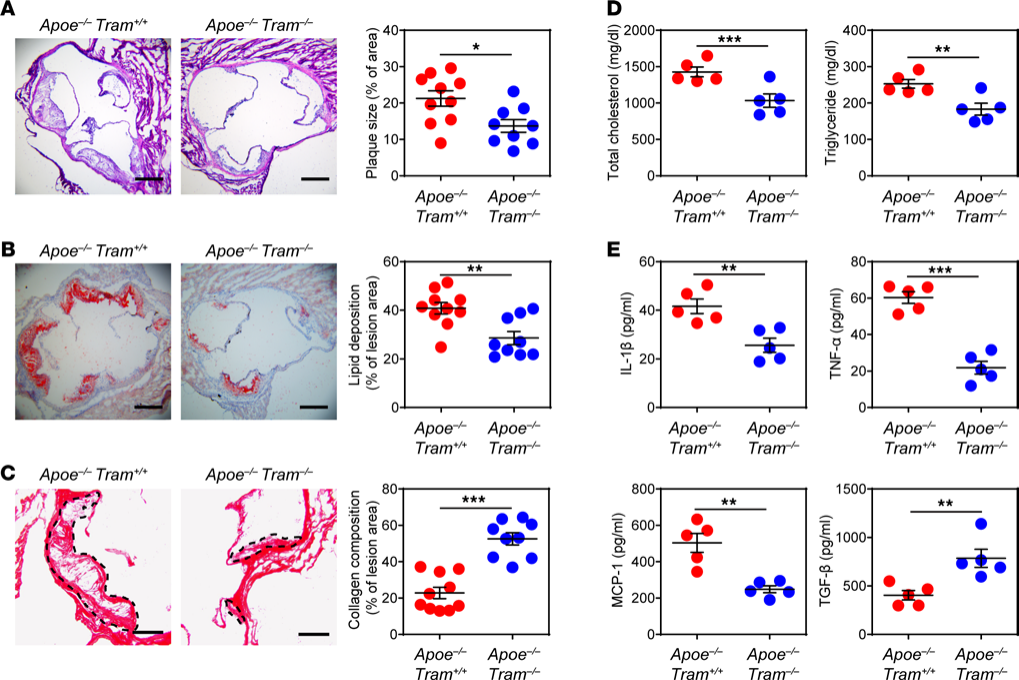
TRAM deficiency alleviates atherosclerosis development. *Apoe^−/−^ Tram^+/+^* mice and *Apoe^−/−^* *Tram^−/−^* mice were fed with HFD for 8 weeks. (**A**) Representative images of H&E-stained atherosclerotic lesions and quantification of plaque size demonstrated as the percentage of lesion area within aortic root area. Scale bar: 300 μm. (**B**) Representative images of Oil Red O–stained atherosclerotic plaques and quantification of lipid deposition within lesion area. Scale bar: 300 μm. (**C**) Representative images of Picrosirius red–stained atherosclerotic plaques and quantification of collagen content within lesion area. Scale bar: 100 μm. (**D**) Determination of total cholesterol and triglyceride levels in the plasma. (**E**) Determination of circulating IL-1β, TNF-α, MCP-1, and TGF-β levels by ELISA. Data are representative of 3 independent experiments, and error bars represent means ± SEM. *P < 0.05, **P < 0.01, and ***P < 0.001; Student’s 2-tailed *t* test (*n* = 5 to 10 for each group).

To determine the contribution of TRAM to the development of atherosclerosis, we generated *Apoe^−/−^* *Tram^−/−^* mice, which were fed with high-fat diet (HFD) for 8 weeks. As compared with HFD-fed *Apoe^−/−^ Tram^+/+^* mice, HFD-fed *Apoe^−/−^* *Tram^−/−^* mice exhibited significantly decreased size of atherosclerotic plaques as evidenced by H&E staining (Figure 1A), diminished lipid deposition in the plaques as shown by Oil Red O staining (Figure 1B), as well as reduced overall lesion area in the aorta (Supplemental Figure 1A; supplemental material available online with this article; https://doi.org/10.1172/jci.insight.149651DS1). In contrast, the collagen content in atherosclerotic lesions was significantly elevated in *Apoe^−/−^* *Tram^−/−^* mice as compared with *Apoe^−/−^ Tram^+/+^* mice, indicating enhanced plaque stability (Figure 1C). The plasma levels of total cholesterol, free cholesterol, and triglyceride were also significantly reduced in *Apoe^−/−^* *Tram^−/−^* mice as compared with *Apoe^−/−^ Tram^+/+^* mice (Figure 1D and Supplemental Figure 1B). Moreover, *Apoe^−/−^* *Tram^−/−^* mice had lower levels of circulating proinflammatory mediators, such as IL-1β, TNF-α, and monocyte chemoattractant protein-1 (MCP-1) but had a higher level of antiinflammatory cytokine TGF-β (Figure 1E). In agreement with an earlier study using the LDL receptor–deficient (*Ldlr*-deficient) animal model with bone marrow transfer (25), our data confirm that TRAM deficiency potently alleviates atherogenesis in atherosclerosis-prone mice accompanied by attenuated inflammation.

### TRAM deficiency inhibits inflammatory monocyte polarization in atherosclerotic mice.

**Figure 2:**
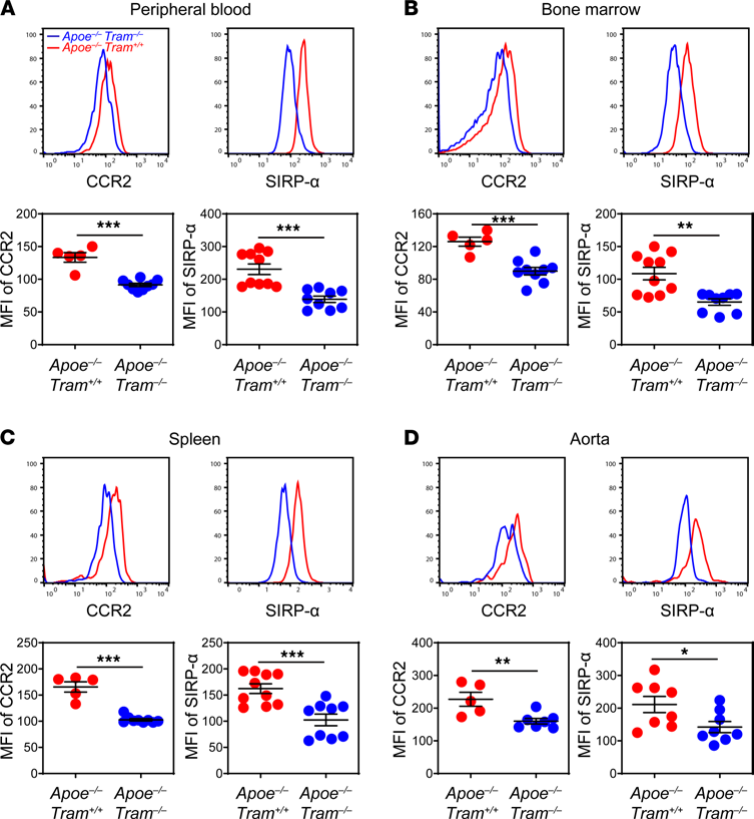
TRAM deficiency reduces monocyte inflammatory state in atherosclerotic mice. *Apoe^−/−^ Tram^+/+^* mice and *Apoe^−/−^* *Tram^−/−^* mice were fed with HFD for 8 weeks. Surface expressions of CCR2 and SIRP-α on CD11b^+^Ly6C^++^ monocytes in the peripheral blood (**A**), BM (**B**), spleen (**C**), and aorta (**D**) were examined by flow cytometry. Data are representative of 2 independent experiments, and error bars represent means ± SEM. *P < 0.05, **P < 0.01, and ***P < 0.001; Student’s 2-tailed *t* test (*n* = 5 to 10 for each group).

Monocytes/macrophages play a leading role in the initiation and development of atherosclerosis, and their proinflammatory polarization is tightly associated with atherosclerosis pathogenesis. Previous studies indicate that murine polarized classical Ly6C^++^ and intermediate Ly6C^+^ inflammatory monocytes (as well as their human equivalents) are responsible for infiltrating inflamed tissues (5, 26) and involved in the progression of atherosclerosis (27, 28). In contrast, the nonclassical Ly6C^lo^ monocytes routinely patrol the vasculature and maintain homeostasis (10). We therefore examined whether TRAM deficiency may affect the polarization of inflammatory monocytes in atherosclerotic mice. After feeding with HFD for 8 weeks, peripheral blood (Figure 2A), bone marrow (BM) (Figure 2B), spleens (Figure 2C), and aortas (Figure 2D) were harvested from *Apoe^−/−^* *Tram^−/−^* mice as well as control *Apoe^−/−^ Tram^+/+^* mice to evaluate the polarization status of inflammatory Ly6C^++^ and Ly6C^+^ monocytes. Relevant inflammatory markers expressed on Ly6C^++^ and Ly6C^+^ monocytes responsible for modulating monocyte recruitment (CCR2) and efferocytosis (SIRP-α) were measured and quantified (Figure 2). CCR2 is a pivotal chemokine receptor mediating the recruitment of monocytes to inflamed sites (29), and SIRP-α is a regulatory protein that enhances proinflammatory signaling in myeloid cells and suppresses efferocytosis (30). We observed that TRAM deficiency contributed to a significant reduction of CCR2 and SIRP-α expressions on Ly6C^++^ monocytes (Figure 2). A similar pattern was also observed on Ly6C^+^ monocytes showing reduced expression of CCR2 and SIRP-α in monocytes harvested from *Apoe^−/−^* *Tram^−/−^* mice as compared with *Apoe^−/−^ Tram^+/+^* mice (Supplemental Figure 2). Consistent with this observation, we further observed that the frequencies of inflammatory monocytes, particularly Ly6C^++^ monocytes, were significantly reduced by approximately 50% in the peripheral blood and spleen of *Apoe^−/−^* *Tram^−/−^* mice (Supplemental Figure 3). Our data complement a previous finding with the *Ldlr^−/−^* atherosclerotic model showing reduced infiltration of inflammatory monocytes within the aortic plaques due to myeloid deficiency of TRAM (25). Our results documenting reduced expression of CCR2 and SIRP-α on inflammatory monocytes due to TRAM deficiency further provide a mechanistic basis underlying reduced pathogenesis of atherosclerosis in TRAM-deficient mice.

### TRAM facilitates low-grade inflammatory monocyte polarization through SRC-mediated activation of STAT1 and STAT5.

**Figure 3:**
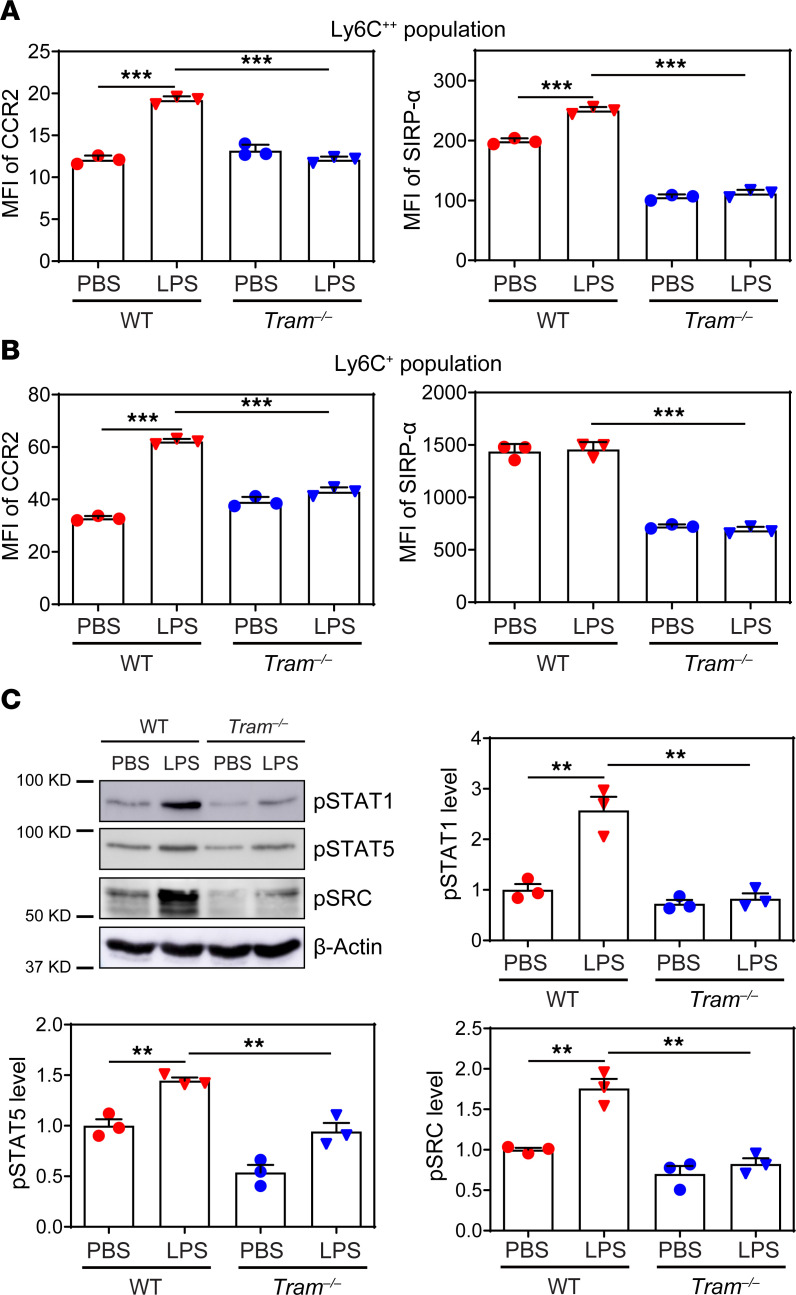
TRAM deficiency attenuates inflammatory polarization of BMMs in vitro. BM cells from WT C57 BL/6 mice and *Tram^−/−^* mice were cultured with M-CSF (10 ng/mL) in the presence of PBS or super-low-dose LPS (100 pg/mL) for 5 days. Surface expressions of CCR2 and SIRP-α on CD11b^+^Ly6C^++^ BMMs (**A**) and CD11b^+^Ly6C^+^ BMMs (**B**) were examined by flow cytometry. (**C**) The phosphorylation of STAT1, STAT5, and SRC in BMMs was examined by Western blotting, and relative levels were normalized to β-actin. Data are representative of 3 independent experiments, and error bars represent means ± SEM. **P < 0.01, and ***P < 0.001; 1-way ANOVA (*n* = 3 for each group).

Next, we sought to examine the molecular mechanism underlying the proinflammatory polarization of monocytes mediated by TRAM. We adopted an in vitro culture system of BM-derived monocytes (BMMs) with M-CSF, capable of robustly expanding classical Ly6C^++^ and intermediate Ly6C^+^ inflammatory monocytes in vitro, upon 5-day incubation with subclinical low-dose LPS (100 pg/mL) (2, 4). Cultured monocytes are loosely attached, can be readily detached via gentle aspiration, express inflammatory monocyte Ly6C^+^/Ly6C^++^ markers, and do not express mature macrophage marker CD71 (31) (Supplemental Figure 6). Previous independent studies reported that similarly cultured monocytes can maintain proliferative/differentiation potential (32, 33). Wild-type (WT) and *Tram^−/−^* BMMs were treated with super-low-dose LPS (100 pg/mL) for 5 days as we described (4). We focused on comparing CCR2 and SIRP-α level between WT and *Tram^−/−^* BMMs. Flow cytometry analyses revealed that prolonged LPS treatment significantly upregulated the expressions of CCR2 and SIRP-α on the surface of WT Ly6C^++^ BMMs. In a sharp contrast, low-dose LPS failed to induce the expression of CCR2 and SIRP-α on Ly6C^++^ BMMs from *Tram^−/−^* mice. Further, the baseline level of SIRP-α in *Tram^−/−^* cells (PBS treatment) was significantly lower than that in WT counterparts (Figure 3A). A similar trend was also observed in Ly6C^+^ BMMs, whose proinflammatory polarization induced by super-low-dose LPS was significantly diminished by TRAM deficiency (Figure 3B). Further, we observed that LPS failed to induce the expansion of the Ly6C^++^ monocyte population in *Tram^−/−^* BMMs as compared with WT BMMs, which readily expanded the Ly6C^++^ population following LPS culture (Supplemental Figure 4). Consistent with in vivo data, our in vitro data reveal that TRAM deficiency reduced proinflammatory polarization of BMMs in vitro.

STAT1 and STAT5, which are key transducers of the TLR4 signaling cascade, mediate the expression of proinflammatory mediators such as CCR2 in myeloid cells (34, 35). We therefore examined the activation status of these key transcription factors by Western blot and flow cytometry and found that LPS treatment led to increased phosphorylation of STAT1 and STAT5 in WT BMMs as compared with *Tram^−/−^* BMMs (Figure 3C and Supplemental Figure 5). We also compared the activation of c-SRC, an upstream signaling molecule that can directly phosphorylate STATs, between WT and *Tram^−/−^* BMMs. Similarly, LPS failed to induce SRC phosphorylation in *Tram^−/−^* BMMs (Figure 3C and Supplemental Figure 5). Together, our data reveal that TRAM is a key signaling node responsible for the low-grade inflammatory monocyte polarization through SRC-mediated activation of STAT1/5.

### TRAM-deficient monocytes are reprogrammed into a resolving state with elevated expression of CD200R in vitro and in vivo.

**Figure 4:**
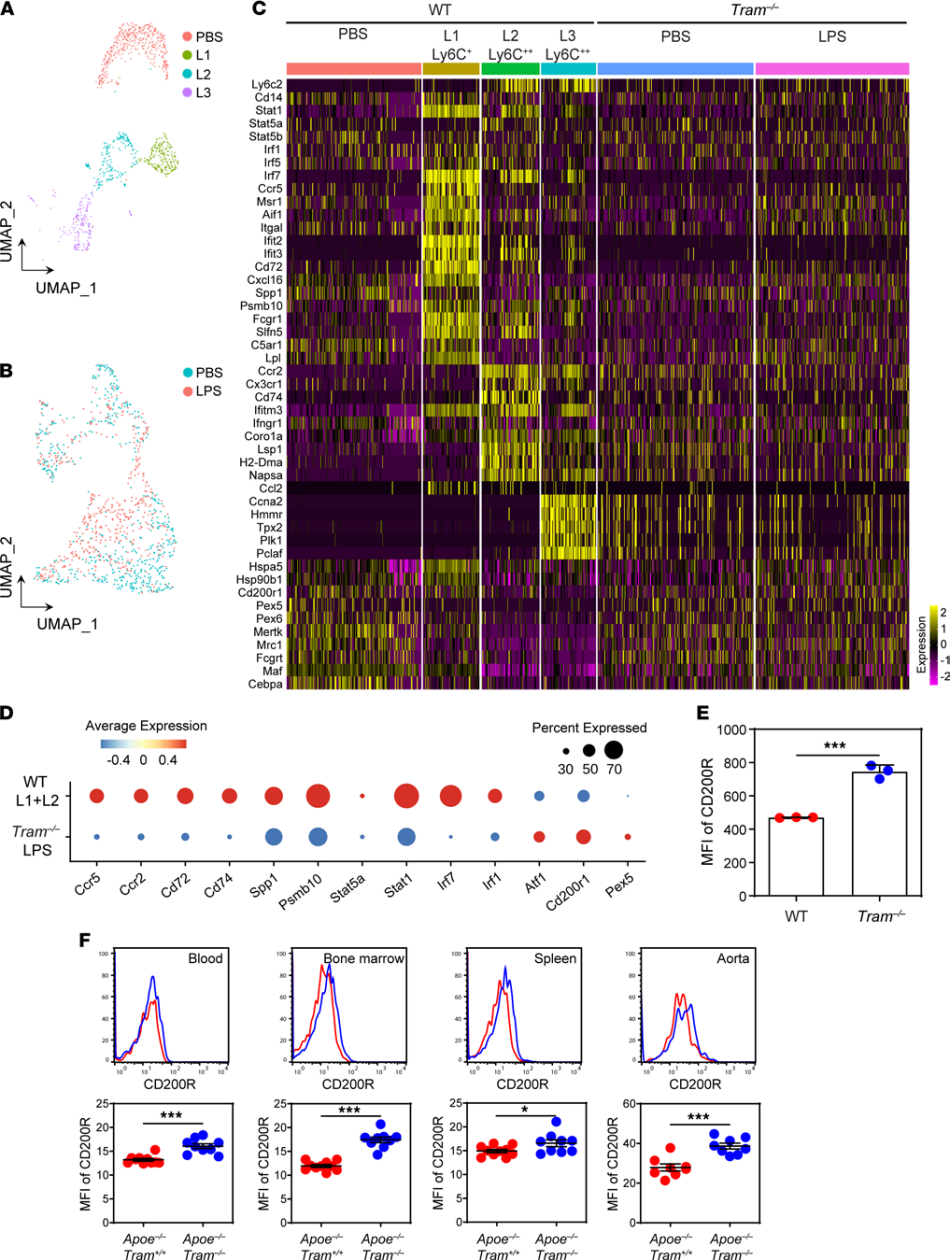
TRAM deficiency gives rise to resolving monocytes with elevated expression of CD200R both in vitro and in vivo. (**A** and **B**) Dimensionality reduction and clustering through uniform manifold approximation and projection (UMAP) of the scRNA-Seq data from WT (**A**) or TRAM monocytes (**B**) challenged with either PBS or 100 pg/mL LPS as we described in Methods. (**C**) Heatmaps showing representative genes differentially expressed in different clusters of monocytes challenged with subclinical-dose LPS. (**D**) Dot plot comparison of representative genes differentially expressed among LPS-treated WT versus *Tram^−/−^* monocytes. (**E**) BM cells from WT C57 BL/6 mice and *Tram^−/−^* mice were cultured with M-CSF (10 ng/mL) for 5 days. The surface expression of CD200R was analyzed and quantified by flow cytometry. (**F**) *Apoe^−/−^ Tram^+/+^* mice and *Apoe^−/−^* *Tram^−/−^* mice were fed with HFD for 8 weeks. Surface expression of CD200R on CD11b^+^Ly6C^++^ monocytes in the peripheral blood, BM, spleen, and aorta was examined by flow cytometry. Data are representative of 2 independent experiments, and error bars represent means ± SEM. *P < 0.05, and ***P < 0.001; Student’s 2-tailed *t* test (*n* = 3 to 10 for each group).

Given the significant role that TRAM plays during monocyte polarization, we further performed single-cell sequencing (scRNA-Seq) analysis of WT and *Tram^−/−^* monocytes challenged with a subclinical dose of endotoxin. Murine inflammatory monocytes are known to adopt at least 2 subpopulations in vitro and in vivo (intermediate CD11bLy6C^+^ and classical CD11bLy6C^++^, respectively). Our scRNA-Seq analyses reveal that WT monocytes persistently challenged with subclinical-dose LPS largely clustered into 2 subpopulations labeled as M_L1_ and M_L2_ (Figure 4, A–C), corresponding to the Ly6C^+^ and Ly6C^++^ monocytes, respectively, confirming our previous studies that subclinical-dose LPS preferentially primes and expands inflammatory monocytes. Confirming our flow cytometry analysis of key cellular protein targets, both Ly6C^+^ M_L1_ and Ly6C^++^ M_L2_ populations expressed higher levels of key inflammatory mediators, such as *Ccr2*, *Ccr5*, *Spp1*, *Cd72,* and *Cd74*, as well as signature inflammatory transcription factors, such as *Stat1*, *Stat5,* *Irf1*, *Irf5*, *Irf7*, and *Aif1*, as compared with naive monocytes. Polarized inflammatory Ly6C^+^ M_L1_ and Ly6C^++^ M_L2_ monocytes by subclinical low-dose endotoxin also preferentially expressed interferon-stimulated genes (*Ifit2*, *Ifit3*, *Ifitm3*, *Ifngr1*, etc.) (Figure 4C), resembling recently identified key features of inflammatory arterial monocyte/macrophage subsets collected from experimental atherosclerotic mice (36). Elevated expression of *Cd74* and *Aif1* has also been reported in inflammatory monocytes/macrophages collected from atherosclerotic plaques of human patients (37, 38). Although both subsets exhibit similar low-grade inflammatory features, Ly6C^+^ M_L1_ monocytes have higher expression of *C5ar1*, *Ccr5,* *Cd72*, *Spp1*, and *Aif1*, while Ly6C^++^ M_L2_ monocytes have higher expression of *Cd74*, *Ccr2*, and *Ly6c*. In addition to the low-grade inflammatory characteristics, elevated *Spp1* expression observed in the our M_L1_ subset was recently documented on aortic intima monocytes/macrophages promoting foam cell formation (39–41). The Ly6C^++^ M_L2_ monocytes better resemble the inflammatory monocytes/macrophages observed in vivo with higher expression of CCR2 (42). On the other hand, we found that super-low-dose LPS potently reduced the expression of selected antiinflammatory mediators such as *Cd200r*, as well as key genes involved in pexophagy, such as *Pex5* (Figure 4C). Intriguingly, our scRNA-Seq revealed an additional separate Ly6C^++^ monocyte subset (M_L3_) (Figure 4C). Compared with the classical inflammatory CD14^+^Ly6C^++^ monocytes, the Ly6C^++^ M_L3_ cells were less inflammatory, expressed less CD14, and possessed proliferative genes based on scRNA-Seq and Gene Ontology (GO) gene enrichment analysis (Figure 4C and Supplemental Figures 6 and 7). Our data complement previous studies that reported the presence of a certain less defined subset of Ly6C^++^ monocytes in vivo, which may be antiinflammatory and have limited proliferative potential (43, 44).

We further performed flow cytometry–based analyses and confirmed the presence of CD14^hi^Ly6C^++^ and CD14^lo^Ly6C^++^ monocytes (Supplemental Figure 8). We found that LPS treatment preferentially expanded the population of inflammatory CD14^hi^Ly6C^++^ classical monocytes by 3-fold (Supplemental Figure 8). In contrast, *Tram^−/−^* monocytes constitutively exhibited reduced levels of inflammatory CD14^hi^Ly6C^++^ population as compared with control WT monocytes (Supplemental Figure 8). LPS failed to expand the inflammatory CD14^hi^Ly6C^++^ population in *Tram^−/−^* monocytes. On the other hand, the percentages of CD14^lo^Ly6C^++^ population comparing WT and *Tram^−/−^* monocytes were comparable and were not significantly altered by LPS. Our scRNA-Seq analyses further confirmed LPS failed to polarize *Tram^−/−^* monocytes and showed that PBS or LPS-treated *Tram^−/−^* monocytes clustered together (Figure 4B). We compared representative genes among WT and *Tram^−/−^* monocytes stimulated with LPS. As shown in Figure 4D, as compared with the inflammatory clusters of WT monocytes challenged with LPS (M_L1_ and M_L2_), *Tram^−/−^* monocytes failed to express low-grade inflammatory mediators, such as *Ccr2*, *Ccr5,* *Cd74,* *Cd72*, and *Spp1*, as well as key transcription factors, such as *Irf1,* *Irf7,* *Stat1,* and *Stat5*.

In contrast, *Tram^–/–^* monocytes constitutively expressed higher levels of antiinflammatory mediator *Cd200r* as well as pexophagy molecule *Pex5*. CD200R, an inhibitory receptor involved in reducing mitogen-activated protein kinase signaling, is crucial for sustaining immune homeostasis and counteracting monocyte/macrophage activation (45, 46). Given its potential significance in resolving inflammation, we further examined the protein levels of CD200R comparing WT and *Tram^−/−^* monocytes in vitro and in vivo. We observed that the resting levels of CD200R in *Tram^−/−^* monocytes were significantly higher as compared with WT monocytes cultured in vitro (Figure 4E). Furthermore, we observed that the levels of CD200R in *Apoe^−/−^* *Tram^−/−^* monocytes harvested from blood, BM, spleen, and aorta were all significantly higher as compared with those harvested from *Apoe^−/−^ Tram^+/+^* mice (Figure 4F). Our data revealed that TRAM deficiency not only blocked the polarization of low-grade inflammatory monocytes induced by subclinical-dose LPS but also gave rise to elevated expression of antiinflammatory resolving mediator CD200R.

### TRAM-deficient monocytes exhibit enhanced pexophagy and reduced intracellular ROS.

**Figure 5:**
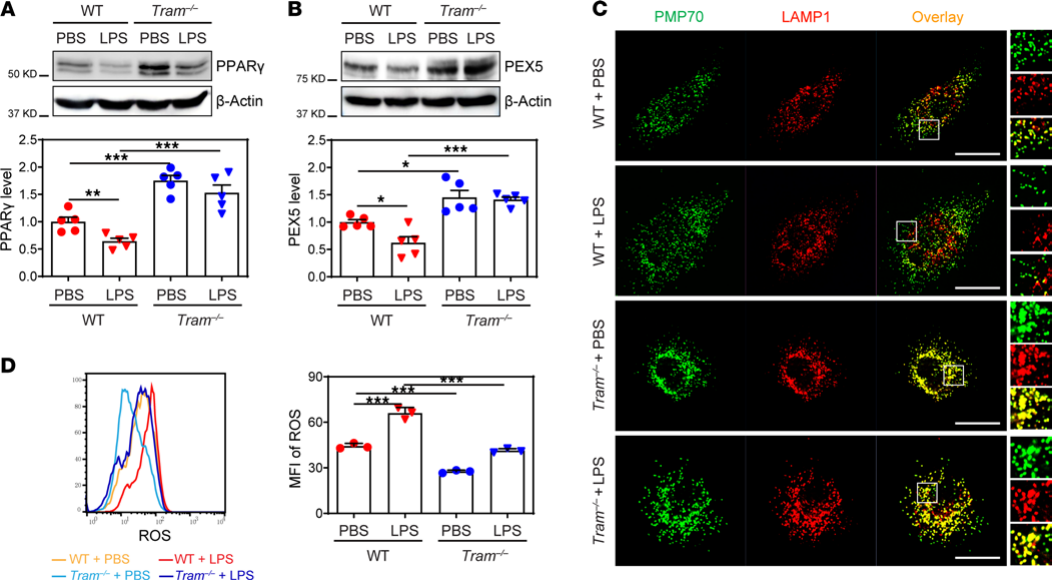
TRAM-deficient resolving monocytes exhibit enhanced peroxisome homeostasis. BM cells from WT C57 BL/6 mice and *Tram^−/−^* mice were cultured with M-CSF (10 ng/mL) in the presence of PBS or super-low-dose LPS (100 pg/mL) for 5 days. (**A** and **B**) Protein levels of PPARγ (**A**) and PEX5 (**B**) were examined by Western blotting, and relative expressions were normalized to β-actin. (**C**) BMMs were stained with anti-PMP70 and anti-LAMP1 antibodies, and the localization of peroxisomes and lysosomes was examined by confocal microcopy. Scale bars: 10 μm. Inset original magnification, 400×. (**D**) BMMs were labeled with CellROX, and ROS levels were quantified by flow cytometry. Data are representative of 3 independent experiments, and error bars represent means ± SEM. *P < 0.05, **P < 0.01, and ***P < 0.001; 1-way ANOVA (*n* = 3 for each group).

It has been reported that intracellular ROS can activate SRC (47) and that compromised fusion of lysosomes with peroxisomes can lead to the accumulation of intracellular ROS (48, 49). Given our finding that pexophagy mediator *Pex5* was reduced in monocytes by subclinical-dose LPS and that TRAM deficiency restored the expression of Pex5, we thus tested whether TRAM deficiency may restore the disrupted peroxisome homeostasis induced by subclinical-dose LPS. To test this, we further quantified the protein levels of crucial molecules involved in the normal pexophagy process, including PEX5 as well as its inducer, PPARγ. Consistent with the scRNA-Seq analysis, we observed that super-low-dose LPS reduced the expression of PEX5 and PPARγ protein levels in WT monocytes (Figure 5, A and B). In contrast, *Tram^−/−^* BMMs constitutively expressed significantly higher PPARγ and PEX5 proteins as compared with WT cells (Figure 5, A and B, and Supplemental Figure 9). Next, we examined peroxisome homeostasis with a focus on its ability to communicate with lysosome. For WT BMMs, LPS treatment potently disrupted peroxisome-lysosome fusion as compared with PBS treatment. In contrast, *Tram^−/−^* monocytes exhibited constitutively elevated fusion of peroxisome and lysosome as compared with WT monocytes (Figure 5C). Further, LPS failed to cause disruption of pexophagy in *Tram^−/−^* monocytes as compared with WT monocytes treated with LPS (Figure 5C). Because altered peroxisome homeostasis affected ROS generation, we determined the levels of intracellular ROS in BMMs through staining with an ROS-specific fluorescent probe followed by flow cytometry. WT BMMs programmed with LPS exhibited significantly elevated ROS level as compared with control WT cells cultured with PBS. In contrast, *Tram^−/−^* monocytes exhibited constitutively less ROS as compared with WT cells (Figure 5D). Collectively, our data unveil a mechanism of TRAM-dependent monocyte/macrophage polarization. With chronic subclinical endotoxemia, TRAM is involved in monocyte inflammatory polarization, at least partially through promoting peroxisome dysfunction and intracellular ROS accumulation, which subsequently activate c-SRC and STATs to alter the phenotype and function of monocytes/macrophages into a proinflammatory state conducive for exacerbated atherosclerosis.

### TRAM-deficient BMMs propagate attenuated inflammatory polarization to neighboring cells partially through elevated CD200R.

**Figure 6:**
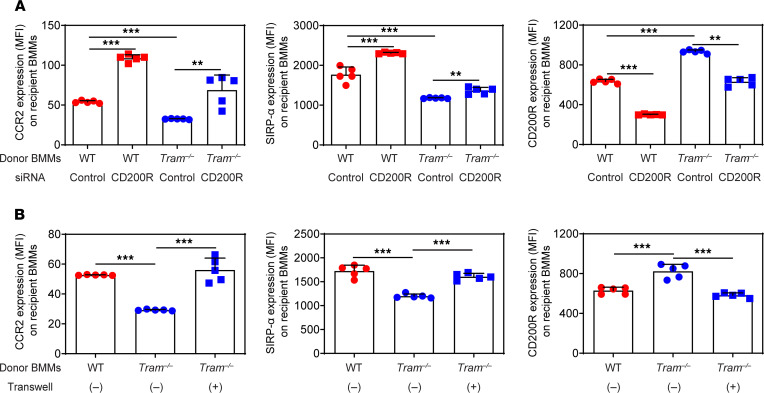
TRAM-deficient BMMs propagate its resolving nature to neighboring monocytes through CD200R. (**A**) BMMs prepared from WT C57 BL/6 mice and *Tram^−/−^* mice, which were both CD45.2^+^, were transfected with CD200R siRNA or control siRNA with Lipofectamine. The CD45.2^+^ donor cells were then cocultured with recipient BMMs prepared from B6 SJL mice, which were CD45.1^+^, for 2 days. Surface expressions of CCR2, SIRP-α, and CD200R on CD45.1^+^ recipient BMMs were examined by flow cytometry. (**B**) Donor BMMs were prepared from WT C57BL/6 mice and *Tram^−/−^* mice, and recipient BMMs were prepared from B6 SJL mice. The donor cells were either directly cocultured with recipient cells or cultured in the upper chamber of a Transwell insert with recipient cells in the lower chamber. After 2 days, surface expressions of CCR2, SIRP-α, and CD200R on CD45.1^+^ recipient BMMs were examined by flow cytometry. Data are representative of 3 independent experiments, and error bars represent means ± SEM. **P < 0.01, and ***P < 0.001; 1-way ANOVA (*n* = 5 for each group).

The immunological synapse formed between CD200R and its ligand, CD200, can mediate intercellular communication among myeloid cells, potentially propagating the antiinflammatory outcomes (46). Based on our finding that TRAM deficiency caused marked elevation of CD200R on the surface of monocytes in vivo and in vitro, we further tested the hypothesis that the increased CD200R *Tram^−/−^* monocytes may propagate inflammation resolution to neighboring cells. To test this, we established a coculture system, in which WT or *Tram^−/−^* BMMs (CD45.2^+^) were used as donor cells to incubate together with recipient BMMs from B6 SJL mice (CD45.1^+^). Before coculture, the donor cells were transfected with either CD200R siRNA or control siRNA, and flow cytometry analysis validated that CD200R expression on the surface of donor BMMs was significantly diminished by CD200R siRNA (Supplemental Figure 10). After coculture for 2 days, the surface phenotype of CD45.1^+^ recipient BMMs was examined, with representative inflammatory molecules (CCR2, SIRP-α) and resolving molecule CD200R as the readouts for polarization. The recipient BMMs cocultured with *Tram^−/−^* donor cells exhibited significantly reduced CCR2 and SIRP-α expressions but increased CD200R expression as compared with those cocultured with WT donor cells, indicating that *Tram^−/−^* BMMs were able to transduce their antiinflammatory resolving phenotype to the neighboring cells. Importantly, CD200R knockdown in *Tram^−/−^* donor cells resulted in marked alleviation of recipient cell polarization (Figure 6A). To further determine whether cell-cell contact may be involved for the polarization of neighboring cells, we placed Transwell inserts to physically separate donor and recipient cells in the coculture system. In contrast to the direct coculture system, *Tram^−/−^* donor cells failed to reduce the expression of CCR2 and SIRP-α on recipient WT BMMs in the Transwell culture system without direct contact. *Tram^−/−^* donor cells also failed to restore the expression of CCR2 on recipient WT BMMs in the Transwell culture system (Figure 6B). Our data suggest that *Tram^−/−^* monocytes may potently propagate the resolving phenotype to neighboring cells through CD200R-mediated intercellular communication.

### Administration of TRAM-deficient monocytes in vivo potently reduces atherosclerosis.

**Figure 7:**
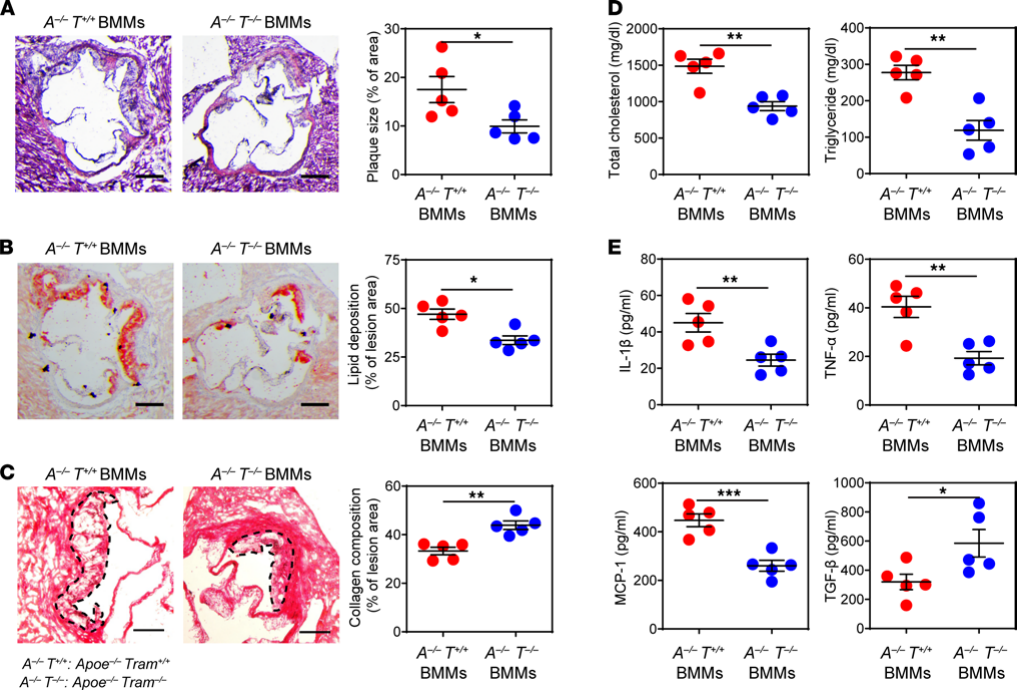
Transfusion of *Tram^−/−^* resolving monocytes ameliorates atherosclerosis. Recipient *Apoe^−/−^ Tram^+/+^* mice were first fed with HFD for 4 weeks. BMMs prepared from *Apoe^−/−^ Tram^+/+^* mice or *Apoe^−/−^* *Tram^−/−^* mice were adoptively transferred by intravenous injection to HFD-fed recipient mice (3 × 10^6^ cells per mouse) once a week for an additional 4 weeks. Samples were collected 1 week after the last BMM transfer. (**A**) Representative images of H&E-stained atherosclerotic lesions and quantification of plaque size demonstrated as the percentage of lesion area within aortic root area. Scale bar: 300 μm. (**B**) Representative images of Oil Red O–stained atherosclerotic plaques and quantification of lipid deposition within lesion area. Scale bar: 300 μm. (**C**) Representative images of Picrosirius red–stained atherosclerotic plaques and quantification of collagen content within lesion area. Scale bar: 100 μm. (**D**) Determination of total cholesterol and triglyceride levels in the plasma. (**E**) Determination of circulating IL-1β, TNF-α, MCP-1, and TGF-β levels by ELISA. Data are representative of 2 independent experiments, and error bars represent means ± SEM. *P < 0.05, **P < 0.01, and ***P < 0.001; Student’s 2-tailed *t* test (*n* = 5 for each group).

Based on our results that TRAM deficiency potently reprograms monocytes into a resolving phenotype with propagating potential, we next tested whether TRAM-deficient resolving monocytes may exert antiatherosclerotic effects when transfused in vivo. *Apoe^−/−^ Tram^+/+^* mice were fed with HFD for 4 weeks and then transfused weekly for an additional 4 weeks with BMMs cultured from *Apoe^−/−^* *Tram^−/−^* mice or control *Apoe^−/−^ Tram^+/+^* mice. The recipient mice were supplemented with HFD during the whole process of the experiment, allowing the development of atherosclerosis. One week after the final cell transfer, the recipient mice were sacrificed for analysis. We observed that mice receiving the transfer of *Tram^−/−^* BMMs had over 40% decrease in plaque sizes and around 30% reduction in the lipid deposition area within atherosclerotic lesions as compared with the mice transfused with control BMMs (Figure 7, A and B, and Supplemental Figure 11A). Picrosirius red staining revealed that administration of *Apoe^−/−^* *Tram^−/−^* BMMs led to significantly higher levels of plaque collagen than *Apoe^−/−^ Tram^+/+^* BMMs (Figure 7C). Plasma cholesterol and triglyceride levels were also substantially decreased in the mice transferred with *Apoe^−/−^* *Tram^−/−^* BMMs as compared with those transferred with *Apoe^−/−^ Tram^+/+^* BMMs (Figure 7D and Supplemental Figure 11B). In addition, we measured key inflammatory mediators in the experimental mice and found that the mice administrated with *Apoe^−/−^* *Tram^−/−^* BMMs had significantly lower plasma levels of IL-1β, TNF-α, and MCP-1 and significantly increased level of TGF-β (Figure 7E). Together, our data revealed that adoptive transfer of *Tram^−/−^* monocytes can effectively alleviate atherosclerosis progression and promote tissue homeostasis in vivo, suggesting a promising approach of immune cell therapy with resolving monocytes for treating atherosclerosis.

## Discussion

In this study, we demonstrate the reprogramming of resolving monocytes through TRAM deletion, which enabled not only the reduction of inflammatory mediators but also the elevation of resolving antiinflammatory mediators. Single-cell sequencing analyses coupled with mechanistic examination revealed constitutively enhanced pexophagy and cellular homeostasis due to TRAM deficiency that underlie the generation of resolving monocytes. Our study further shows the intriguing potential of resolving monocytes in propagating homeostasis and reducing the pathogenesis of atherosclerosis.

Our findings complement and extend previous studies in the emerging field of innate immune memory and programming dynamics. Dependent upon the signal strength and duration of innate challenges, monocytes/macrophages are known to adopt distinct activation states, such as priming and tolerance (50). While prolonged stimulation of monocytes with higher doses of LPS can lead to compensatory antiinflammatory tolerance (51, 52), persistent challenge with subclinical super-low-dose LPS fails to induce tolerance (53). Subclinical super-low-dose endotoxemia is prevalent in humans and experimental animals with chronic diseases due to compromised mucosal barrier and has been associated with the pathogenesis of atherosclerosis (4, 19, 54). Although the principle of priming was appreciated, previous studies were limited in scope and precision due to the lack of single-cell analysis. Capitalizing on these findings, our current study further clarified the low-grade inflammatory landscape of primed monocytes subjected to the persistent challenge of pathologically relevant subclinical-dose LPS with single-cell precision. Our data define the generation of intermediate Ly6C^+^ M_L1_ and classical Ly6C^++^ M_L2_ inflammatory monocytes in vitro by sustained challenge of subclinical low-dose endotoxin, consistent with the in vivo expansion of these monocyte subsets under atherosclerotic conditions. Consistent with previous observations (37, 38, 42), the Ly6C^+^ M_L1_ and Ly6C^++^ M_L2_ monocyte clusters induced by subclinical dose LPS primarily expressed inflammatory mediators involved in chemotaxis and adhesion (e.g., *Ccr2*, *Ccr5*, *Cd74*) as well as key transcription factors mediating inflammatory responses, such as STAT1/5, IRF1/7, and AIF1, without compensatory expression of antiinflammatory mediators. In contrast to higher dose LPS, which can induce antiinflammatory tolerance, subclinical-dose LPS potently suppressed selected antiinflammatory mediators, such as CD200R, within these inflammatory monocyte clusters.

Our single-cell analysis also reveals several features of low-grade inflammatory monocytes programmed by subclinical-dose LPS. First, the general expression profiles included genes involved in lysosome functions as well as interferon-related genes, without robust induction of classical acute response cytokines such as *Tnfα*, reflecting their low-grade inflammatory polarization nature. We also observed that the polarizing effects of subclinical-dose LPS were absent in *Tram^−/−^* monocytes, consistent with the preferential role of TRAM adaptor in facilitating interferon regulatory factor–related gene expressions (55, 56). Second, subclinical-dose LPS potently suppressed genes involved in peroxisome homeostasis, such as *Pex5*. This correlated with elevated intracellular ROS in LPS-polarized monocytes, which may further activate redox-sensitive SRC kinase responsible for the activation of STAT1/STAT5. Our data also identify TRAM as an important modulator involved in peroxisome homeostasis, since *Tram^−/−^* monocytes expressed constitutively higher levels of Pex5 and failed to downregulate Pex5 upon LPS challenge. Third, our single-cell analysis better characterizes the intermediate Ly6C^+^ M_L1_ and classical CD14^hi^Ly6C^++^ M_L2_ subsets of low-grade inflammatory monocytes generated by prolonged stimulation with subclinical-dose LPS. Although both subsets shared some general features as discussed above, there were some distinct differences. M_L1_ monocytes had preferential expression of *Ccr5*, *Cd72*, *Trem2*, and *Spp1*, while M_L2_ monocytes had higher expression of *Ccr2*, *Cd74*, and *Ly6c*. Although mechanisms responsible for their divergent polarization are not clear, their generation may represent coordinated low-grade inflammatory events relevant to the pathogenesis of chronic diseases such as atherosclerosis. Indeed, recent single-cell analyses with monocytes collected in vivo from atherosclerotic animals have identified distinct clusters of monocytes, with some clusters sharing features with inflammatory signatures such as *Ccr2* as well as interferon-stimulated genes, as well as clusters poised for foam cell formation with signature genes of *Spp1* and *Trem2* (41, 42). Recent studies with monocytes/macrophages collected from human atherosclerotic patients also reveal elevated expression of Cd74 (38). Our in vitro model with prolonged subclinical-dose LPS challenge presents a well-defined system for future mechanistic clarification of the ontogeny and stability of primed low-grade inflammatory monocytes, transition among these subsets, as well as their functional implications.

Our data confirmed that TRAM is an important mediator for the expansion of inflammatory monocytes, and TRAM deficiency can block LPS-induced generation of inflammatory CD14^hi^Ly6C^++^ monocytes. *Tram^–/–^* monocytes still retained the CD14^lo^Ly6C^++^ monocyte subset with elevated expression of cell cycle–related genes, which may correlate with an in vivo–observed Ly6C^++^ subset with antiinflammatory effects (43, 44). Our mechanistic observations reveal that TRAM facilitates the low-grade inflammatory monocyte polarization through reducing PPARγ-mediated PEX5 expression, leading to the disruption of pexophagy and induction of ROS. Our data further reveal that TRAM deletion not only attenuated the low-grade inflammatory monocyte polarization but also gave rise to a stable resolving monocyte state with constitutively elevated expression of PEX5 and PPARγ, as well as antiinflammatory mediator CD200R. *Tram*^–/–^ monocytes are constitutively antiinflammatory, which may explain the substantially reduced pathogenesis of atherosclerosis in *Tram*^–/–^ mice, likely due to the presence of resolving monocytes with elevated CD200R observed in vivo. Our data revealing distinct subsets of Ly6C^++^ monocytes (CD14^hi^ vs. CD14^lo^) are consistent with previous phenotypic observations reporting functional heterogeneity of Ly6C^++^ monocytes during the pathogenesis and regression of atherosclerosis (28, 43, 44). Future studies are required to better clarify their ontogeny, relative stability and plasticity, as well as functional involvements during the pathogenesis of atherosclerosis. Future systems studies with integrated genetic and pharmacological tools are also needed to better define signaling molecules in addition to TRAM involved in context-dependent reprogramming of monocytes.

The significance of resolving leukocytes in treating chronic diseases has been increasingly recognized (57–59). Contributing to this concept, our data reveal that resolving monocytes due to TRAM deletion can potently suppress the expression of chemokine receptor CCR2, thus preventing the recruitment of monocytes to atherosclerotic plaques. We demonstrate that resolving monocytes not only exhibit reduced CCR2 expression but also can proactively propagate its resolving nature to neighboring monocytes, potentially through cell-cell communication mediated by CD200R. At the translational level, our data reveal the novel potential of transfusing resolving monocytes with TRAM deletion in alleviating the pathogenesis of atherosclerosis.

We realize that the pathogenesis of atherosclerosis is highly complex, and the effects of monocytes/macrophages are not solely limited to tissue infiltration and inflammatory modulation. We and others previously reported that LPS treatment also leads to the downregulation of cholesterol efflux genes, such as ABCA1 (60, 61). Thus, elevated infiltration of CD14^hi^Ly6C^++^ inflammatory monocytes within atherosclerotic plaque tissues may further compromise cholesterol processing and alter plasma lipid composition. Inflamed circulating monocytes may also interact with endothelial cells and contribute to compromised vasculature integrity (10, 62). Future studies are needed to better define the relative contributions of various subsets of Ly6C^+^ inflammatory monocytes as well as patrolling Ly6C^lo^ monocytes during the complex process of atherosclerosis pathogenesis.

Collectively, our data characterize the nature of low-grade inflammatory monocytes primed by subclinical low-dose endotoxin relevant to the pathogenesis of atherosclerosis and define TRAM as a key bifurcation switch facilitating the polarization of low-grade inflammatory monocytes in vitro and in vivo. Deletion of TRAM potently gave rise to resolving monocytes potentially capable of propagating inflammation resolution and regression of atherosclerosis. Our observations help lay a conceptual foundation for future immunotherapies employing reprogrammed resolving monocytes to treat atherosclerosis.

## Methods

### Mice.

WT C57BL/6 mice, *Apoe^−/−^* mice, and B6 SJL mice were purchased from The Jackson Laboratory. *Tram^–/–^* mouse colony was provided by Holger Eltzschig (University of Texas Houston Medical School, Houston, Texas, USA). *Apoe^–/–^ Tram^–/–^* mice were obtained by crossing *Apoe^–/–^* mice with *Tram^–/–^* mice, all with C57BL/6 background. The mice were bred and maintained under specific pathogen–free conditions in the Association for Assessment and Accreditation of Laboratory Animal Care International–accredited animal facility at Virginia Tech. Both male and female mice between 8 and 10 weeks of age were used for experiments, and no sex-specific effects were observed.

### HFD feeding, adoptive transfer, and sample collection.

Age- and sex-matched *Apoe^−/−^ Tram^+/+^* mice and *Apoe^–/–^ Tram^–/–^* mice were fed with HFD for 8 weeks followed by sample collection. Adoptive transfer of cells was conducted as described previously (4). BM cells isolated from *Apoe^−/−^ Tram^+/+^* mice and *Apoe^–/–^ Tram^–/–^* mice were cultured in complete RPMI 1640 medium (containing 10% FBS, 2 mM l-glutamine, and 1% penicillin/streptomycin) supplemented with M-CSF (10 ng/mL) for 5 days, yielding BMMs. Age- and sex-matched recipient *Apoe^−/−^ Tram^+/+^* mice were fed with HFD for 4 weeks to induce atherosclerosis development, then were transfused with 3 × 10^6^ in vitro–generated BMMs through intravenous injection once a week for 4 weeks. The mice were continuously fed with HFD during the adoptive transfer regimen. One week after the last cell transfer, the mice were sacrificed, and tissues were harvested for subsequent analyses.

### Analyses of atherosclerotic lesions.

Histological analyses of atherosclerotic lesions were performed as previously described (4, 63, 64). Briefly, freshly frozen and optimal cutting temperature compound–embedded proximal aortic sections (10 μm) were fixed in 4% neutral buffered formalin followed by H&E staining. Oil Red O staining was performed using a kit (Newcomer Supply), and collagen staining was performed using the Picrosirius Red Stain Kit (Polysciences) according to the manufacturers’ instructions. For harvesting the proximal aorta, the mice were anesthetized with 1% isoflurane and perfused with 4% paraformaldehyde. The tissues surrounding the aortic tree were carefully removed under a dissection microscope. The samples were observed under a light microscope. The percentages of total lesion area, lipid deposition, and collagen composition were calculated.

### ELISA and determination of plasma lipids.

Plasma samples were collected from the mice treated as described above. The levels of IL-1β, TNF-α, MCP-1, and TGF-β were determined with ELISA kits purchased from R&D Systems. Total and free cholesterol levels were quantified with a kit purchased from MilliporeSigma, and triglyceride level was quantified with a kit purchased from BioVision. All assays were performed according to the manufacturers’ instructions.

### Analyses of monocyte phenotype in vivo.

Peripheral blood, BM, spleen, and aorta were harvested from the mice treated as described. Blood, BM, and spleen samples were disassociated with mechanical processes. Aorta samples were prepared according to the protocol described before (65) with modifications. Briefly, aortas were cut into small pieces and then transferred into an enzyme cocktail (in HBSS) containing 450 U/mL collagenase type I (Worthington), 250 U/mL collagenase type XI (MilliporeSigma), 120 U/mL hyaluronidase (MilliporeSigma), and 120 U/mL DNAse (MilliporeSigma) supplemented with 20 mM HEPES (MilliporeSigma). The samples were incubated at 37°C in an automated tissue dissociator (Miltenyi Biotec) for 60 minutes. The processed samples of all tissues were filtered through 70 μm cell strainers to obtain single-cell suspension, and red blood cells were lysed with ACK buffer (Thermo Fisher Scientific). The samples were incubated with anti-CD16/-CD32 antibodies (1:100 dilution, BD Biosciences, no. 553141) to block Fc receptors followed by staining with fluorochrome-conjugated anti-CD11b (1:200 dilution, BioLegend, no. 101226), anti-Ly6G (1:200 dilution, BioLegend, no. 127606), anti-Ly6C (1:200 dilution, BioLegend, no. 128018), anti-CCR2 (1:200 dilution, BioLegend, no. 150628), anti–SIRP-α (1:200 dilution, BioLegend, no. 144014), and anti-CD200R (1:200 dilution, BioLegend, no. 123916) antibodies. Propidium iodide (PI, MilliporeSigma) was added before flow cytometry to label dead cells. The surface phenotype of PI^–^Ly6G^–^CD11b^+^Ly6C^++^ monocytes and PI^–^Ly6G^–^CD11b^+^Ly6C^+^ monocytes was examined using FACSCanto II (BD Biosciences). The data were analyzed using FlowJo (Tree Star).

### Flow cytometry analyses of in vitro–primed BMMs.

BM cells isolated from WT C57BL/6 mice and *Tram^–/–^* mice were cultured in complete RPMI 1640 medium supplemented with M-CSF (10 ng/mL) in the presence of super-low-dose LPS (100 pg/mL) or PBS as described previously (12). Fresh LPS or PBS was added to the cell cultures every 2 days. After 5 days, BMMs were harvested, filtered through 70 μm cell strainers, and incubated with anti-CD16/-CD32 antibodies (1:100 dilution, BD Biosciences, no. 553141) to block Fc receptors. For surface phenotype analyses, the BMMs were stained with fluorochrome-conjugated anti-CD11b (1:200 dilution, BioLegend, no. 101226), anti-Ly6C (1:200 dilution, BioLegend, no. 128018), anti-CCR2 (1:200 dilution, BioLegend, no. 150628), anti–SIRP-α (1:200 dilution, BioLegend, no. 144014), and anti-CD200R (1:200 dilution, BioLegend, no. 123916) antibodies, and PI (MilliporeSigma) was added before flow cytometry. For detection of intracellular molecules, the cells were fixed and permeabilized using a transcription factor phospho buffer set (BD Biosciences), then stained with fluorochrome-conjugated anti–p-STAT1 (1:100 dilution, Cell Signaling Technology, no. 8009), anti–p-STAT5 (1:100 dilution, Thermo Fisher Scientific, no. 11-9010-42), anti–p-SRC (1:100 dilution, Thermo Fisher Scientific, no. 12-9034-42), or anti-PPARγ (1:50 dilution, Bioss Antibodies, no. bs-4590R-A488) antibodies. For detecting PEX5, the fixed and permeabilized cells were stained with primary rabbit anti–mouse PEX5 antibody (1:100 dilution, Proteintech, no. 12545-1-AP) followed by Alexa Fluor 647–conjugated goat anti–rabbit IgG (1:500 dilution, Thermo Fisher Scientific, no. A32733). Intracellular ROS level was determined with the method described previously (63). Briefly, 5 μM CellROX Green Reagent (Thermo Fisher Scientific) was added to BMM cultures 30 minutes before harvesting. The samples were examined using FACSCanto II, and the data were analyzed using FlowJo.

### Immunoblotting.

PBS- or super-low-dose LPS–primed BMMs from WT C57BL/6 mice and *Tram^–/–^* mice were prepared as described before (12). Total protein was extracted with RIPA buffer (Thermo Fisher Scientific) containing a protease inhibitor cocktail (MilliporeSigma) and then subjected to SDS-PAGE and transferred to a polyvinylidene difluoride membrane. The membrane was incubated with blocker (Bio-Rad) at room temperature for 1 hour and then incubated with primary anti-PPARγ (1:500 dilution, Santa Cruz Biotechnology, no. sc-7273), anti-PEX5 (1:500 dilution, Proteintech, no. 12545-1-AP), anti–p-STAT1 (1:500 dilution, Cell Signaling Technology, no. 9177), anti–p-STAT5 (1:500 dilution, Cell Signaling Technology, no. 9359), anti–p-SRC (1:500 dilution, Cell Signaling Technology, no. 2105), or β-actin antibody (1:1000 dilution, Cell Signaling Technology, no. 5125) overnight at 4°C, followed by incubation with HRP-conjugated anti–rabbit IgG (1:1000 dilution, Cell Signaling Technology, no. 7074) or anti–mouse IgG secondary antibody (1:1000 dilution, Cell Signaling Technology, no. 7076) for 1 hour at room temperature. Blots were developed by an ECL detection kit (Thermo Fisher Scientific).

### Single-cell sequencing and analysis.

BMMs harvested from WT C57BL/6 mice and *Tram^–/–^* mice programmed in vitro with either PBS or super-low-dose LPS were prepared as described before (12). cDNA libraries were generated from treated cells by using the 10x Genomics Chromium Single Cell 3′ Reagent Kits (version 3 Chemistry) and sequenced by Novogene on the Illumina HiSeq platform. Approximately 1000 single-cell gel beads in emulsion per sample were prepared. The cDNAs of each sample were amplified for 12 cycles, quantified by Qubit, and quality checked by Bioanalyzer to verify the size distribution of cDNA samples (~400 bp) and subsequently used for library preparation. Indexed library samples following the successful passage of quality control by Tapestation were quantified by KAPA library quantification kit (Universal). Pooled library samples were sequenced by Novogene through paired-end sequencing on Illumina HiSeq 4000 platform, with the read length of 150 bp at each end plus 8 bp i7 index.

Raw sequencing data were analyzed using the Cell Ranger (version 3.1.0) with mouse reference genome and annotation (Cell Ranger reference version 3.3.0, mm10, Ensembl 93) from the 10x Genomics website (https://support.10xgenomics.com/single-cell-gene-expression/software). Cell Ranger (version 3.0.2) was used to perform the alignment and mapping of sequenced reads, as well as the quantification of relative levels of gene expression. Quality control and data normalization were performed using the default pipeline of Seurat (version 3.1.4) in R (66), which filtered out doublets. Cells that had fewer than 200 unique genes were excluded; genes that existed in fewer than 3 cells were also removed. Approximately 2500 cells were retained and merged. Further, a small cluster of neutrophils expressing Ly6g was also excluded. Dimensionality reduction was performed by principal component analysis, and cells were clustered through UMAP analysis. Gene enrichment GO analyses were performed as described (67, 68). The data set was deposited to the NCBI database under Gene Expression Omnibus accession GSE182237.

### Confocal microscopy.

PBS- or super-low-dose LPS–primed BMMs from WT C57BL/6 mice and *Tram^–/–^* mice were prepared as described before, and peroxisome-lysosome fusion was examined by confocal microscopy as previously reported (63). Peroxisomes were stained using SelectFX Alexa Fluor 488 peroxisome labeling kit (Thermo Fisher Scientific) according to the manufacturer’s instruction. Briefly, primed BMMs were rinsed with PBS, fixed with 4% paraformaldehyde, and permeabilized with 0.2% Triton X-100. The samples were blocked with 10% goat serum and stained with primary rabbit anti–mouse PMP70 antibody (1:1000 dilution), followed by staining with Alexa Fluor 488 goat anti–rabbit secondary antibody (1:1000 dilution). Paraformaldehyde, Triton X-100, goat serum, rabbit anti–mouse PMP70 antibody, and Alexa Fluor 488 goat anti–rabbit secondary antibody were all supplied in the kit. After extensive washing with PBS, the cells were then stained with Cy3 anti–LAMP1 antibody (1:1000 dilution; Abcam, no. Ab67283) to label lysosomes. The samples were mounted with antifade mountant (Invitrogen) and observed under an LSM 900 confocal microscope (ZEISS). Images were processed with ZEN lite (ZEISS).

### CD200R knockdown and coculture assay.

BM cells isolated from WT C57BL/6 mice and *Tram^–/–^* mice (CD45.2^+^) were cultured in 6-well plates with complete RPMI 1640 medium supplemented with M-CSF (10 ng/mL). Stock CD200R siRNA (Thermo Fisher Scientific), control siRNA (Thermo Fisher Scientific), and Lipofectamine reagent (Thermo Fisher Scientific) were diluted in Opti-MEM (Thermo Fisher Scientific), and siRNA was mixed with Lipofectamine reagent to transfect BM cultures (25 pmol siRNA/well). After 3 days, the cells were harvested, stained with anti-Ly6G (1:200 dilution, BioLegend, no. 127606) and anti-CD11b (1:200 dilution, BioLegend, no. 101226) antibodies, and labeled with PI. CD11b^+^Ly6G^–^PI^–^ cells were purified with SH800 cell sorter (Sony) to obtain donor BMMs. BM cells isolated from B6 SJL mice (CD45.1^+^) were cultured in 12-well plates (5 × 10^5^ cells/well) with complete RPMI 1640 medium supplemented with M-CSF (10 ng/mL) to serve as recipient cells. After 3 days, floating cells were removed, and adherent BMMs were cocultured with WT or *Tram^–/–^* donor BMMs (5 × 10^5^ cells/well) for an additional 2 days in the presence of M-CSF (10 ng/mL). Then the cells were harvested and stained with anti-CD45.1 (1:200 dilution, BioLegend, no. 110730), anti-CCR2 (1:200 dilution, BioLegend, no. 150628), anti–SIRP-α (1:200 dilution, BioLegend, no. 144014), and anti-CD200R (1:200 dilution, BioLegend, no. 123916) antibodies. The surface phenotype of CD45.1^+^ recipient BMMs was determined with flow cytometry.

### Transwell assay.

BM cells isolated from WT C57BL/6 mice and *Tram^–/–^* mice (CD45.2^+^) were cultured with complete RPMI 1640 medium supplemented with M-CSF (10 ng/mL) for 3 days. The cells were harvested, stained with anti-Ly6G (1:200 dilution, BioLegend, no. 127606) and anti-CD11b (1:200 dilution, BioLegend, no. 101226) antibodies, and labeled with PI. CD11b^+^Ly6G^–^PI^–^ cells were purified with SH800 cell sorter (Sony) to obtain donor BMMs. BM cells isolated from B6 SJL mice (CD45.1^+^) were cultured in 12-well plates (5 × 10^5^ cells/well) with complete RPMI 1640 medium supplemented with M-CSF (10 ng/mL) to serve as recipient cells. After 3 days, floating cells were removed, and Transwell inserts (Corning) were placed in some recipient cell cultures. WT or *Tram^–/–^* donor BMMs were added to the upper chambers of the Transwell inserts (5 × 10^5^ cells/well) or directly added to the recipient BMMs without Transwell inserts (5 × 10^5^ cells/well). After 2 days, the cells were harvested and stained with anti-CD45.1 (1:200 dilution, BioLegend, no. 110730), anti-CCR2 (1:200 dilution, BioLegend, no. 150628), anti–SIRP-α (1:200 dilution, BioLegend, no. 144014), and anti-CD200R (1:200 dilution, BioLegend, no. 123916) antibodies. The surface phenotype of CD45.1^+^ recipient BMMs was determined with flow cytometry.

### Statistics.

Statistical analyses were performed using Prism software (GraphPad). All data are expressed as means ± SEM, and the sample number for each data set is provided in figure legends. Comparisons between 2 groups were performed by using 2-tailed Student’s *t* test, and comparisons among multiple groups were carried out with 1-way ANOVA. *P* < 0.05 was considered statistically significant.

### Study approval.

All experimental procedures were approved by the Intuitional Animal Care and Use Committee of Virginia Tech in compliance with the US NIH *Guide for the Care and Use of Laboratory Animals* (National Academies Press, 2011).

## Author contributions

SG designed experiments, performed studies, analyzed data, generated figures and wrote the manuscript; YZ performed experiments; ZY performed experiments and analyzed the data; RL performed experiments; and LL designed experiments, supervised studies, and wrote the manuscript.

## Supplementary Material

Supplemental data

## Version 1 09/09/2021

In-Press Preview

## Version 2 10/22/2021

Electronic publication
