# Supplementary material for: Resolving monocytes generated through TRAM deletion attenuate atherosclerosis
Source: JCI Insight. 2021 Oct 22;6(20):e149651. doi: 10.1172/jci.insight.149651 (PMC8564896; doi:10.1172/jci.insight.149651)
Supplement: Supplemental data [file jciinsight-6-149651-s066.pdf]

Supplementary Figures

Resolving monocytes generated through TRAM deletion attenuate the pathogenesis of atherosclerosis

Shuo Geng[1], Yao Zhang[1], Ziyue Yi[2], Ran Lu[1], Liwu Li[1,2]*

[1]Department of Biological Sciences, Virginia Tech, Blacksburg, VA24061; [2]Graduate Program of Genetics, Biotechnology and Computational Biology, Virginia Tech, Blacksburg, VA24061

Keywords: Monocyte polarization, Inflammation resolution, atherosclerosis, TRAM, systems dynamics

Running Title: Resolving monocytes alleviate atherosclerosis

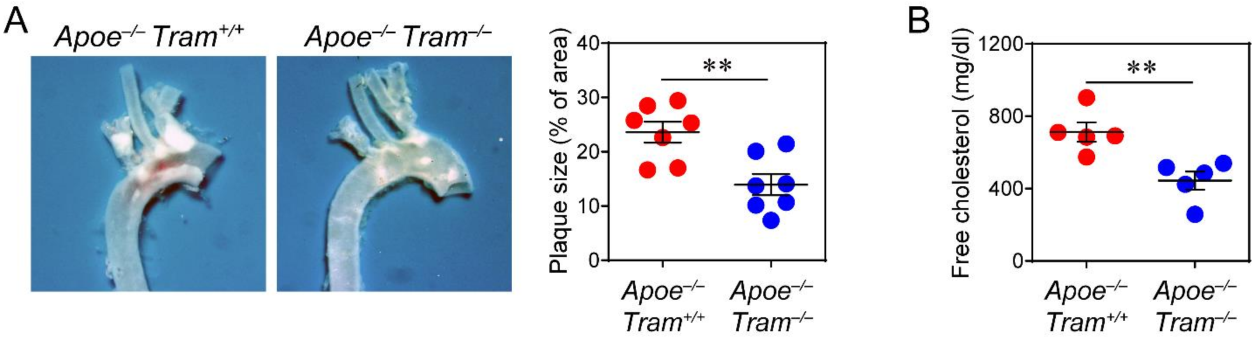

Supplemental Figure 1 (Related to Figure 1). Reduced plaque size and free cholesterol level in TRAM deficient mice. *Apoe*$^{-/-}$ *Tram*$^{+/+}$ mice and *Apoe*$^{-/-}$ *Tram*$^{-/-}$ mice were fed with HFD for 8 weeks. (A) Excised proximal aorta was observed under a microscope, and the percentage of lesion area within the aorta was quantified. (B) Free total cholesterol in the plasma was determined. Data are representative of three independent experiments, and error bars represent means ± SEM. **$P < 0.01$; Student's *t* test ($n = 5$ or 7 for each group).

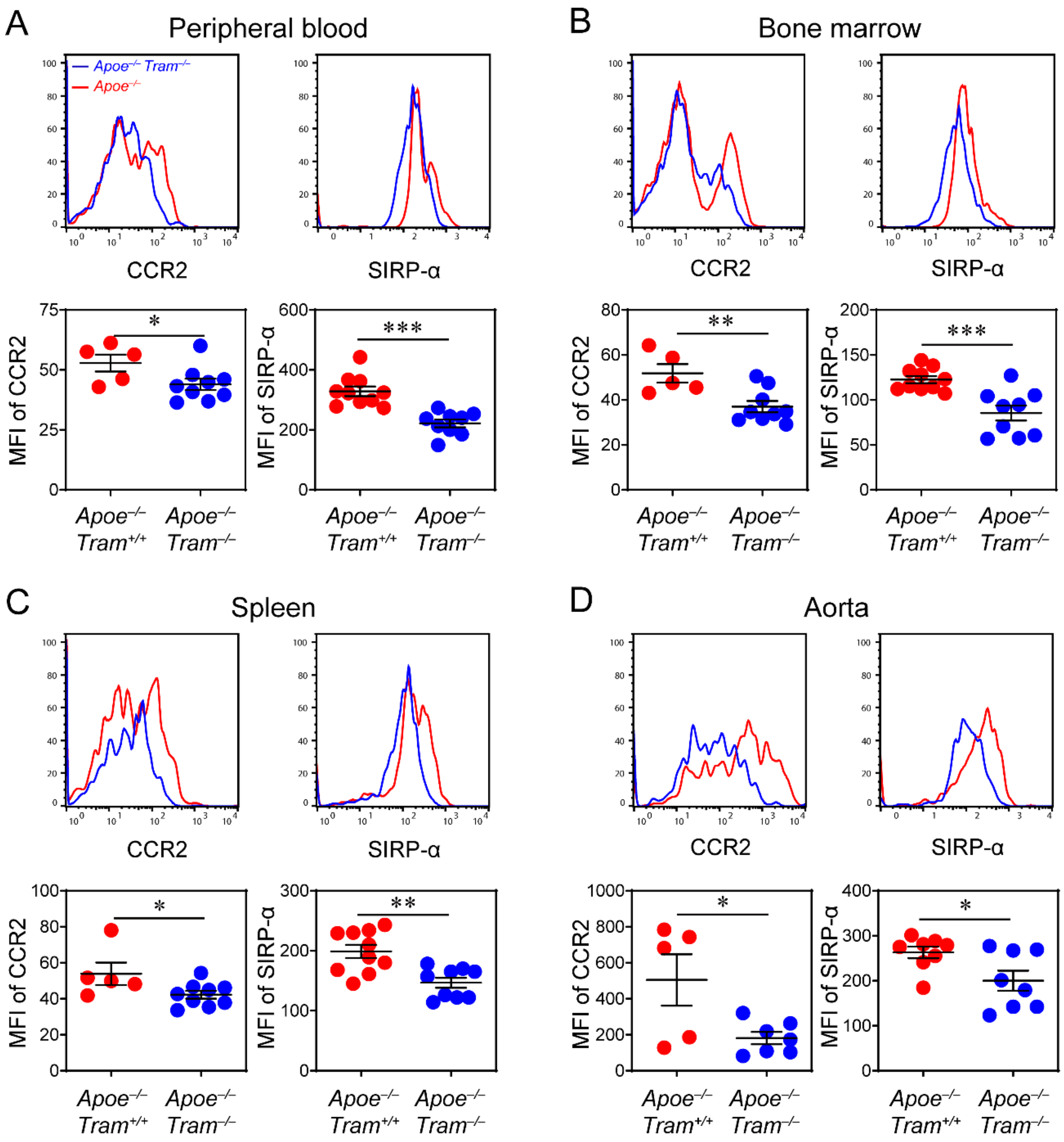

Supplemental Figure 2 (Related to Figure 2). TRAM deficiency attenuates inflammatory state of CD11b⁺/Ly6C⁺ monocytes in atherosclerotic mice. *Apoe*$^{-/-}$ *Tram*$^{+/+}$ mice and *Apoe*$^{-/-}$ *Tram*$^{-/-}$ mice were fed with HFD for 8 weeks. Surface expressions of CCR2 and SIRP-α on CD11b^{+}/Ly6C^{+} monocytes in the peripheral blood (A), bone marrow (B), spleen (C) and aorta (D) were examined by flow cytometry. Data are representative of two independent experiments, and error bars represent means ± SEM. *P < 0.05, **P < 0.01, and ***P < 0.001; Student's t test (n = 5 to10 for each group).

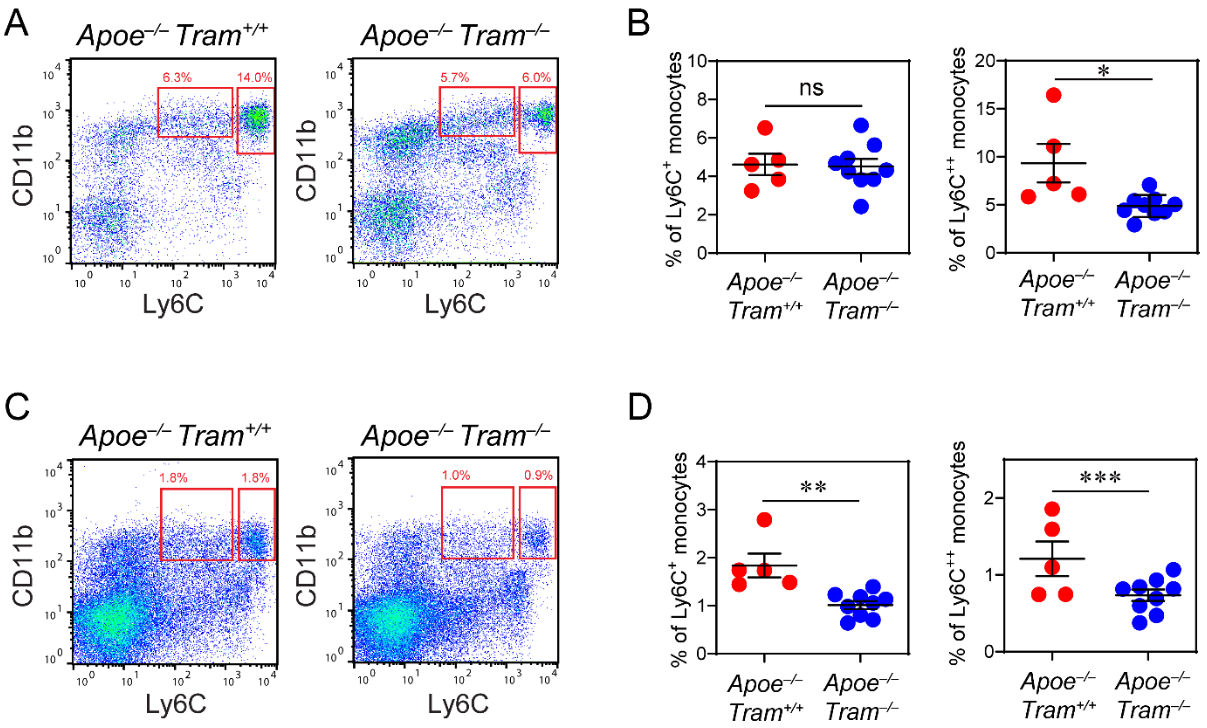

Supplemental Figure 3 (Related to Figure 2). Decreased pro-inflammatory monocytes in TRAM deficient mice. *Apoe*$^{-/-}$ *Tram*$^{+/+}$ mice and *Apoe*$^{-/-}$ *Tram*$^{-/-}$ mice were fed with HFD for 8 weeks. (A and B) Peripheral blood cells were collected, and CD11b^{+}/Ly6C^{++} and CD11b^{+}/Ly6C^{+} monocytes gated within the Ly6G^{-} population were examined by flow cytometry. The frequency of pro-inflammatory monocytes within total leukocytes was quantified. (C and D) Splenocytes were collected, and CD11b^{+}/Ly6C^{++} and CD11b^{+}/Ly6C^{+} monocytes gated within the Ly6G^{-} population were examined by flow cytometry. The frequency of pro-inflammatory monocytes within total leukocytes was quantified. Data are representative of two independent experiments, and error bars represent means ± SEM. *P < 0.05, **P < 0.01, and ***P < 0.001; Student's t test (n = 5 for *Apoe*$^{-/-}$ group, and n = 9 for *Apoe*$^{-/-}$ *Tram*$^{-/-}$ group).

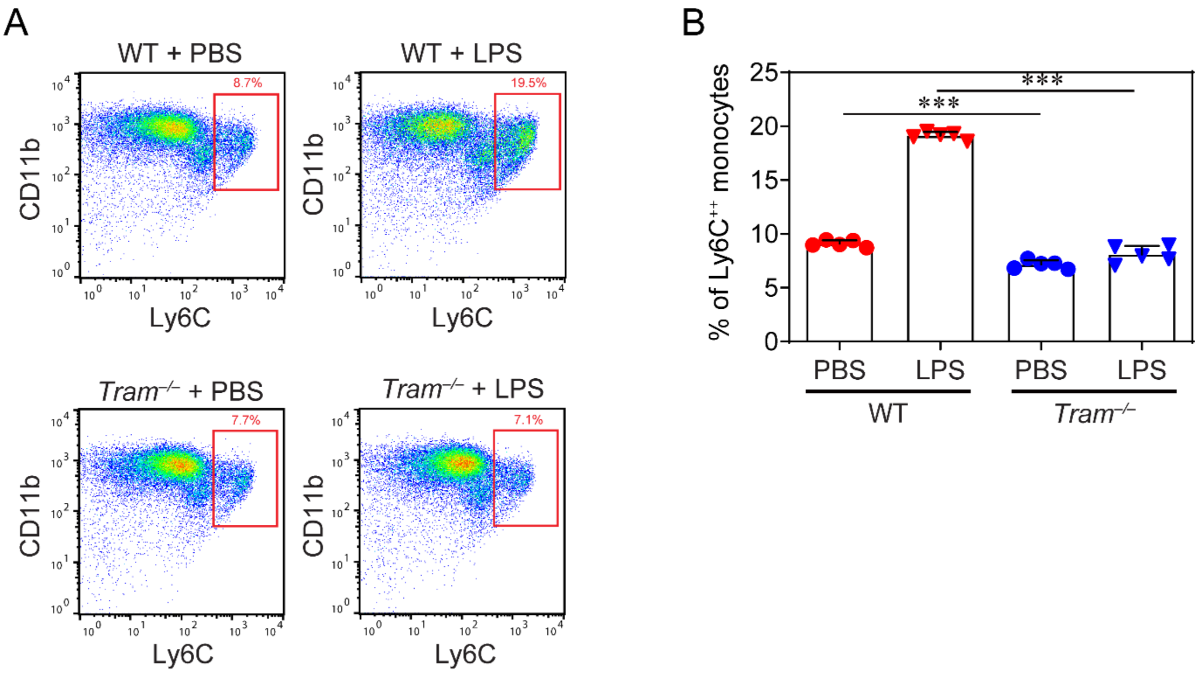

Supplemental Figure 4 (Related to Figure 3). LPS fails to expand Ly6C^{++} population in BMM culture. (A and B) Bone marrow cells from WT C57 BL/6 mice and *Tram*$^{-/-}$ mice were cultured with M-CSF (10 ng/ml) in the presence of PBS or super-low-dose LPS (100 pg/ml) for 5 days. The frequency of CD11b^{+}/Ly6C^{++} BMMs was examined and quantified by flow cytometry. Data are representative of three independent experiments, and error bars represent means ± SEM. ***P < 0.001; one-way ANOVA (n = 5 for each group).

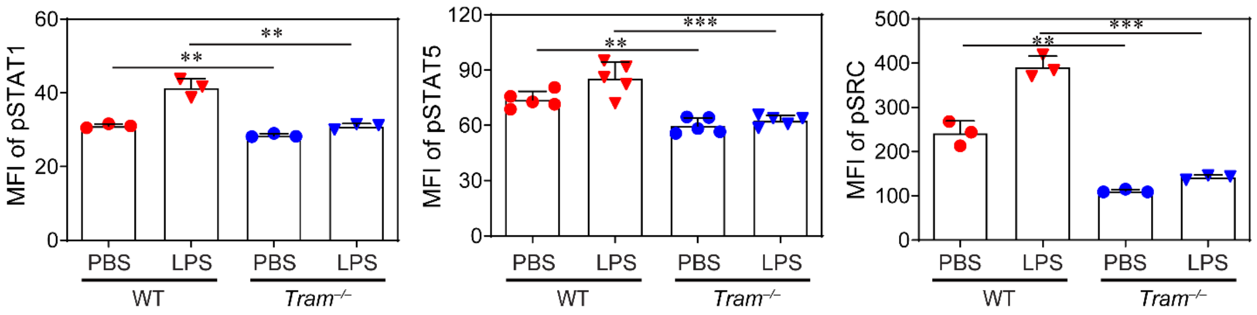

Supplemental Figure 5 (Related to Figure 3). Reduced pSTAT1, pSTAT5 and pSRC levels in TRAM deficient BMMs. Bone marrow cells from WT C57 BL/6 mice and *Tram*$^{-/-}$ mice were cultured with M-CSF (10 ng/ml) in the presence of PBS or super-low-dose LPS (100 pg/ml) for 5 days. The levels of pSTAT1, pSTAT5 and pSRC were analyzed and quantified by flow cytometry. Data are representative of three independent experiments, and error bars represent means ± SEM. $^{**}P < 0.01$, and $^{***}P < 0.001$; one-way ANOVA (n = 3 or 5 for each group).

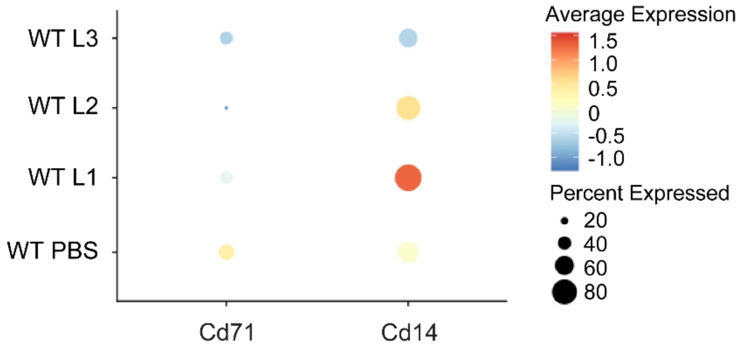

Supplemental Figure 6 (Related to Figure 4). Differential expressions of Cd14 and the lack of Cd71 expression in monocyte subsets. Dot plot comparison of Cd71 and Cd14 expressed among LPS-treated and PBS-treated WT monocytes through scRNAseq analysis.

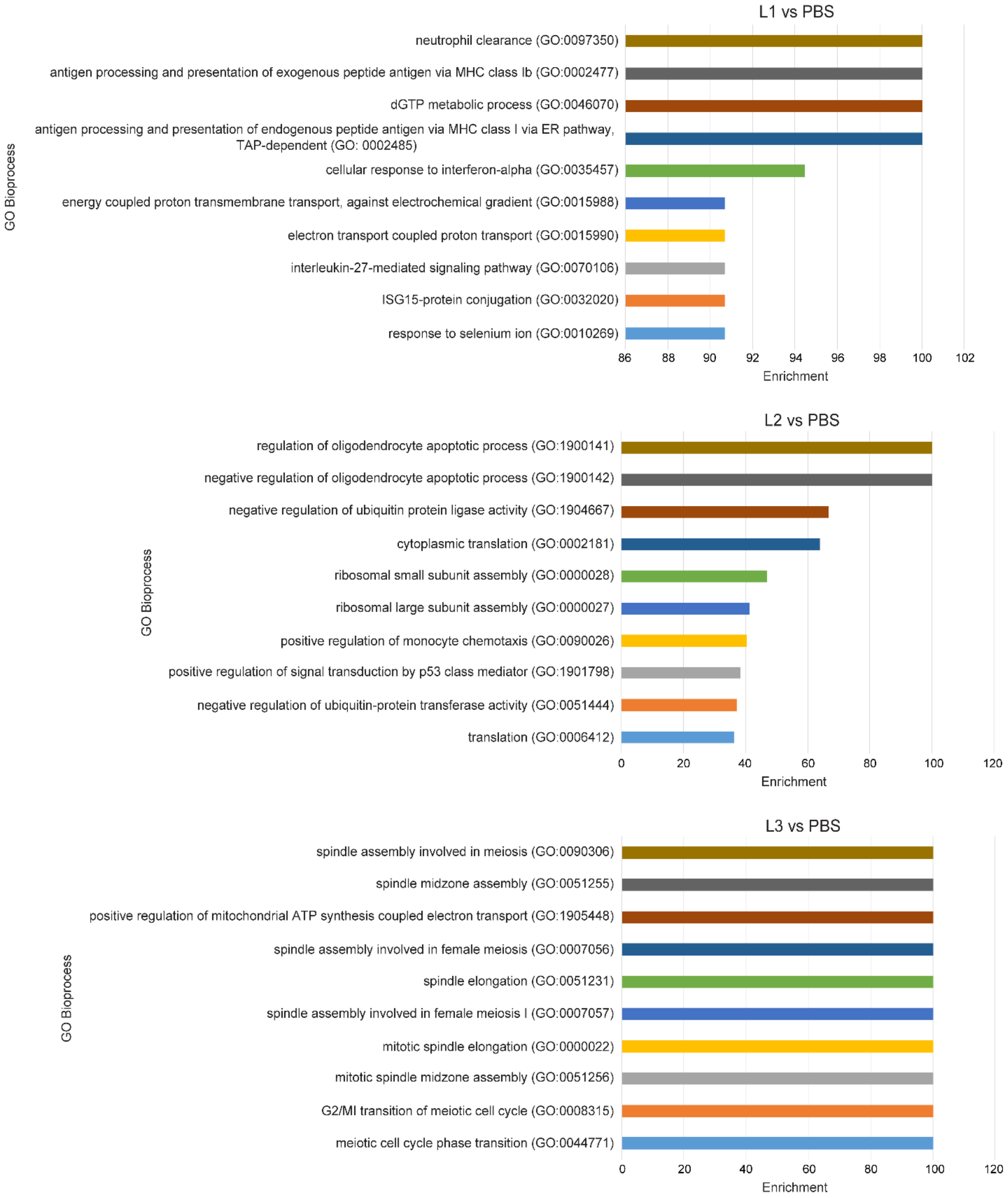

Supplemental Figure 7 (Related to Figure 4). Enrichment analyses of GO biological process categories representing significantly altered genes among LPS-programmed monocyte subsets as compared to PBS control monocytes.

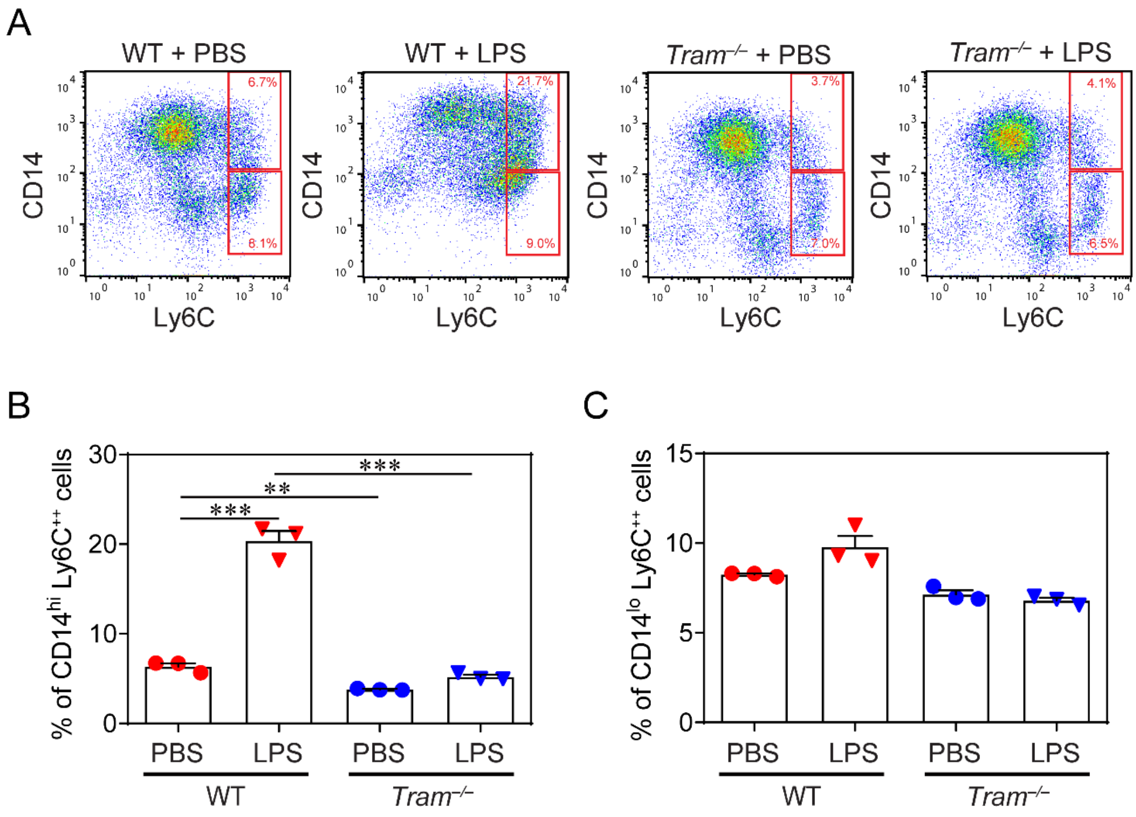

Supplemental Figure 8 (Related to Figure 4). Bone marrow cells from WT C57 BL/6 mice and *Tram*$^{-/-}$ mice were cultured with M-CSF (10 ng/ml) in the presence of PBS or super-low-dose LPS (100 pg/ml) for 5 days. (A) The surface expression of Ly6C and CD14 was examined by flow cytometry. The frequencies of CD14^{hi}/Ly6C^{++} population (B) and CD14^{lo}/Ly6C^{++} population (C) were quantified. Data are representative of three independent experiments, and error bars represent means ± SEM. **$P < 0.01$, and ***$P < 0.001$; one-way ANOVA (n = 3 for each group).

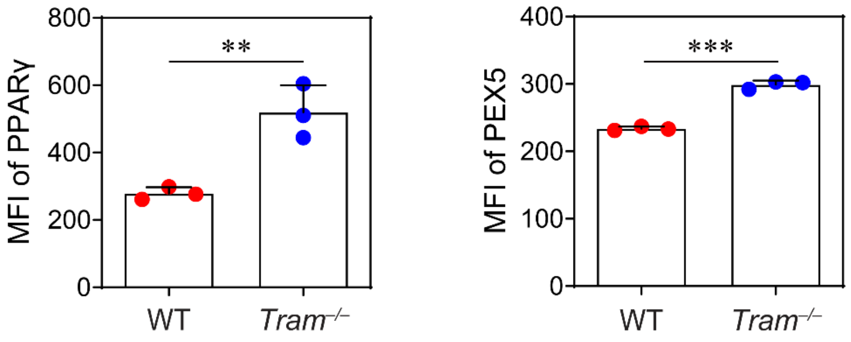

Supplemental Figure 9 (Related to Figure 5). Elevated PPARγ and PEX5 expressions in TRAM deficient BMMs. Bone marrow cells from WT C57 BL/6 mice and *Tram*$^{-/-}$ mice were cultured with M-CSF (10 ng/ml) for 5 days. The levels of PPARγ (A) and PEX5 (B) were analyzed and quantified by flow cytometry. Data are representative of three independent experiments, and error bars represent means ± SEM. $^{**}P < 0.01$, and $^{***}P < 0.001$; Student's t test (n = 3 for each group).

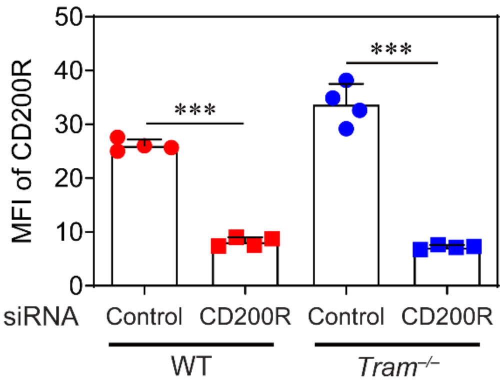

Supplemental Figure 10 (Related to Figure 6). CD200R siRNA knocks down CD200R expression in donor BMMs. BMMs prepared from WT C57 BL/6 mice and *Tram*$^{-/-}$ mice were transfected with CD200R siRNA or control siRNA with Lipofectamine, and surface expression of CD200R was examined by flow cytometry. Data are representative of three independent experiments, and error bars represent means ± SEM. ***$P < 0.001$; one-way ANOVA (n = 4 for each group).

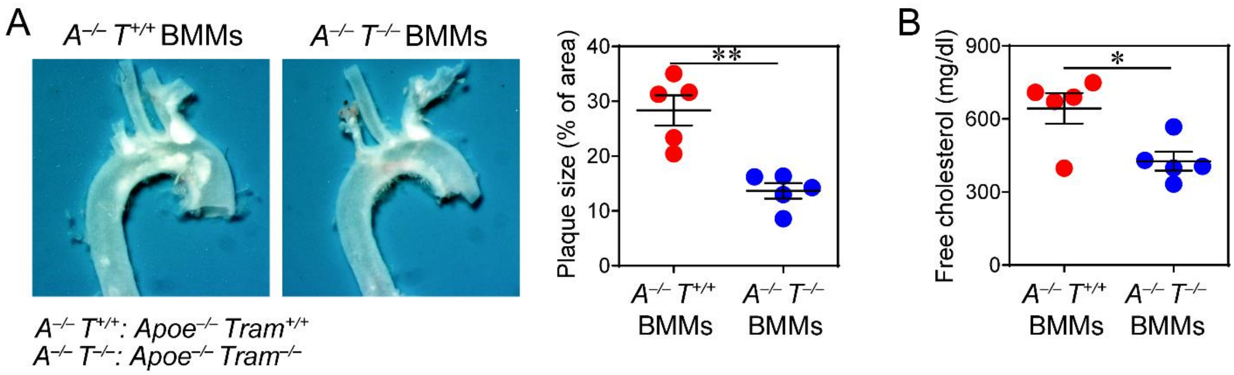

Supplemental Figure 11 (Related to Figure 7). Reduced free cholesterol level in atherosclerotic mice transfused with *Tram*$^{-/-}$ resolving monocytes. Recipient *Apoe*$^{-/-}$ *Tram*$^{+/+}$ mice were first fed with HFD for 4 weeks. BMMs prepared from *Apoe*$^{-/-}$ *Tram*$^{+/+}$ mice or *Apoe*$^{-/-}$ *Tram*$^{-/-}$ mice were adoptively transferred by intravenous injection to HFD-fed recipient mice (3×10^6 cells per mouse) once a week for additional 4 weeks. Samples were collected 1 week after the last BMM transfer. (A) Excised proximal aorta was observed under a microscope, and the percentage of lesion area within the aorta was quantified. (B) Free total cholesterol in the plasma was determined. Data are representative of two independent experiments, and error bars represent means ± SEM. *$P < 0.05$, and **$P < 0.01$; Student's *t* test (n = 5 for each group).
